# Consuming Information Related to COVID-19 on Social Media Among Older Adults and Its Association With Anxiety, Social Trust in Information, and COVID-Safe Behaviors: Cross-sectional Telephone Survey

**DOI:** 10.2196/26570

**Published:** 2021-02-11

**Authors:** Frankie Ho Chun Wong, Tianyin Liu, Dara Kiu Yi Leung, Anna Y Zhang, Walker Siu Hong Au, Wai Wai Kwok, Angie K Y Shum, Gloria Hoi Yan Wong, Terry Yat-Sang Lum

**Affiliations:** 1 Department of Social Work and Social Administration The University of Hong Kong Pokfulam Hong Kong; 2 Sau Po Centre on Aging The University of Hong Kong Pokfulam Hong Kong

**Keywords:** COVID-19, anxiety, social media, infodemic, Hong Kong

## Abstract

**Background:**

COVID-19-related information on social media is overabundant and sometimes questionable, resulting in an “infodemic” during the pandemic. While previous studies suggest social media usage increases the risk of developing anxiety symptoms, how induced anxiety affects attitudes and behaviors is less discussed, let alone during a global pandemic. Little is known about the relationship between older adults using social media during a pandemic and their anxiety, their attitudes toward social trust in information, and behaviors to avoid contracting COVID-19.

**Objective:**

The goal of this study was to investigate the associations between using social media for COVID-19-related information and anxiety symptoms as well as the mediation effect of anxiety symptoms on social trust in information and COVID-safe behaviors among older adults.

**Methods:**

A cross-sectional telephone survey was conducted in Hong Kong between May and August 2020. A rapid warm-call protocol was developed to train social workers and volunteers from participant nongovernmental organizations to conduct the telephone surveys. Questions related to COVID-safe behaviors, social trust in information, social media use, anxiety and depressive symptoms, and sociodemographic information were asked. The number of confirmed COVID-19 cases at the community level was used to account for the risk of contracting COVID-19. Ordinary least squares regressions examined the associations between social media use and anxiety symptoms, and how they were associated with social trust in information and COVID-safe behaviors. Structural equation modeling further mapped out these relationships to identify the mediation effects of anxiety symptoms.

**Results:**

This study collected information regarding 3421 adults aged 60 years and older. Use of social media for COVID-19-related information was associated with more anxiety symptoms and lower social trust in information but had no significant relationship with COVID-safe behaviors. Anxiety symptoms predicted lower social trust in information and higher COVID-safe behaviors. Lower social trust in information was predicted by using social media for COVID-19 information, mediated by anxiety symptoms, while no mediation effect was found for COVID-safe behaviors.

**Conclusions:**

Older adults who rely on social media for COVID-19-related information exhibited more anxiety symptoms, while showing mixed effects on attitudes and behaviors. Social trust in information may be challenged by unverified and contradictory information online. The negligible impact on COVID-safe behaviors suggested that social media may have caused more confusion than consolidating a consistent effort against the pandemic. Media literacy education is recommended to promote critical evaluation of COVID-19-related information and responsible sharing among older adults.

## Introduction

The COVID-19 pandemic is the first in history in which technology and social media have been used on a massive scale to keep people safe, informed, productive, and connected [1]. Although older adults are arguably less tech-savvy and do not use social media as often as younger adults, previous studies suggest that using social media for communication may help maintain relationships and prevent loneliness among older adults while having the potential to enhance health-related knowledge through information seeking [2,3]. However, the overabundance of information, especially misinformation about the COVID-19 pandemic because of its novelty and associated uncertainty, is fueling an infodemic [4]. Since the pandemic is likely to be enduring, its prolonged impact on mental health is anticipated, rendering it an important research and practice priority [5]. Therefore, effort should be made to understand the role of social media use in mental health among older adults during the COVID-19 pandemic.

Exposure to mass media in crises has been associated with anxiety symptoms and distress [6,7]. Meanwhile, the use of social media presents a higher risk of having anxiety disorder [8], where passive information consumption such as reading the news is associated with stronger anxiety symptoms [9,10]. The characteristics of social media and the way older adults consume it may further facilitate the spread of anxiety during a public health crisis such as the COVID-19 pandemic. Most recent evidence suggests an association between social media use and increased anxiety symptoms during the pandemic [11-13]. Researchers suggest that in the epicenter, Wuhan, China, this association could be a result of the fear inflicted by misinformation circulating online, while social media amplified widespread nervousness and worry [14]. Previous experiences during the Korean Middle East Respiratory Syndrome (MERS) outbreak also demonstrated the positive relationship between social media exposure and higher perceived public health risks [15]. Therefore, reliance on social media for acquiring information related to the COVID-19 pandemic introduces a higher chance of being overwhelmed by unverified and contradictory messages that promote the vicious cycle of anxiety.

During the COVID-19 pandemic, adults aged 60 years or older in Hong Kong had a significantly higher risk of exhibiting anxiety symptoms compared to the general population [16]. Challenges are presented to older adults, who may respond to the pandemic and social media differently. Contrary to the primarily unidirectional communication process in traditional media, interactive social media platforms allow users to engage in different modes of health communication [17]. A systematic review showed that previous research seldom distinguished different purposes of social media use among older adults [18]. Active usage, such as sharing or posting personal content, is different in nature from passive usage, such as browsing or seeking information that requires less effort and no need to communicate a self-concept with others in the virtual world [9]. While digital technologies designed for older adults typically stimulate passive usage [2], digital-savvy older adults are developing distinct ways of adopting internet usage, substituting online sources for traditional media for news and information consumption [19]. Passive usage is common among older adults in Hong Kong, of whom 68% use the internet for reading news and 71% listen to or watch multimedia content [20]. While their one-way consumption of information and the social media environment suggest a higher risk of having anxiety symptoms [9,10], more evidence is needed to understand whether using social media for COVID-19 information has a negative impact on older adults during the pandemic.

Worries over physical health are a major source of anxiety-related concern among older adults, where the cognitive phenomenon of worrying is central to their experience [21]. The higher the health risk, the more likely older adults feel anxious. These health-related concerns could be intensified by COVID-19-related media consumption portraying the virus as particularly harmful to the older population [22]. Inconsistent and overwhelming information available on social media exacerbates worries about the pandemic [23]. Therefore, exposure to COVID-19-related information on social media during the pandemic may induce anxiety symptoms [23-25]. It is hypothesized that older adults who use social media for COVID-19-related information will exhibit more anxiety symptoms.

Anxiety may contribute to pandemic-related attitudes. One of the impacts of anxiety on attitudes is its association with social distrust [26]. Recent research in the context of COVID-19 demonstrates that using social media as an information source is associated with conspiracy beliefs [27]. Anxious about contradictory information online, older adults using social media may show more disbelief toward what is told. As a result, the attitudes in trusting the information about COVID-19 from the social circles around these older adults may be affected. While trust in information may also affect how much older adults use a particular media platform, this study is particularly interested in how the infodemic may affect older adults. Especially when the literature suggested that information on social media may prompt anxiety, the implication of investigating the mediation effect from using social media for COVID-19 information on social trust in information is to unveil the effects of media usage and the ways older adults are getting to know the world. It is hypothesized that older adults for whom social media is their main source of COVID-19-related information have lower social trust in information, mediated by anxiety symptoms.

While the information on social media mediates personal mitigation strategies, anxiety may prompt COVID-safe behaviors, including handwashing and social distancing [28]. Meanwhile, excessive anxiety may lead to maladaptive COVID-safe behaviors, such as panic purchasing, or nonadherence to public health recommendations [29]. Given the immense public health risk, anxious older adults should be encouraged to adopt more COVID-safe behaviors. Wearing face masks, frequent hand hygiene, and social distancing by staying home were three COVID-safe behaviors suggested by infectious disease experts in Hong Kong [30]. Both recommendations of wearing face masks and frequent hand hygiene were widely followed by residents. For example, 98.8% of all residents wore face masks in early 2020 before it was mandated by the government [31]. However, the social distancing recommendation was observed by fewer people for various reasons (eg, work, shopping, exercise, and meals with friends). Therefore, we chose social distancing, measured by the number of days each week older adults ventured into the community, as a proxy for COVID-safe behavior in this study. It is hypothesized that older adults who use social media as their source of COVID-19-related information will reduce social contact by reducing time spent in the community, mediated by anxiety symptoms.

**Figure 1:**
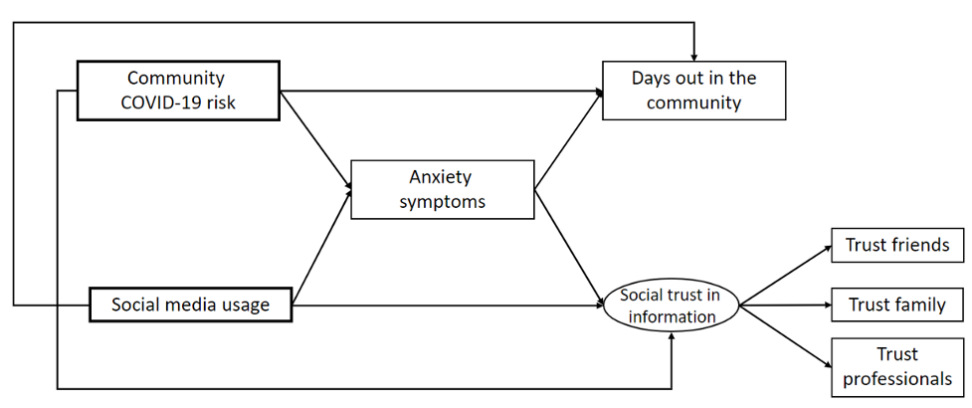
Theoretical framework.

Anxiety symptoms, social trust in information, and time spent in the community may be influenced by the objective risk of contracting COVID-19. In order to distinguish the effects of the actual risk from the effects of the perceptions acquired via social media, the hypothesized theoretical framework put the risk of contracting COVID-19 as another independent factor (see Figure 1).

The specific focus of this study was to investigate the effects of consuming public health–related information via social media on anxiety among the older population during a public health crisis. Some of the most recent studies investigating the impact of information sources on confidence in coping with COVID-19 [32], behaviors [33], and beliefs about health-related information [34] have primarily used internet-based questionnaires to survey their participants. Since, in this study, we were interested in evaluating older people, some of whom may be less engaged in digital communication, an online survey would present difficulties in reaching them and could create sampling bias. Therefore, a telephone survey was chosen to capture a more diverse sample. To address the gap in the current literature, this study investigated how using social media for COVID-19 information among older adults during the pandemic was associated with anxiety symptoms and how the two contribute to social trust in information and COVID-safe behaviors.

## Methods

### Study Design and Sampling

A cross-sectional telephone survey was conducted during the COVID-19 outbreak in Hong Kong between May and August 2020; participants included adults aged 60 years and older using services provided by community elderly centers and community mental wellness centers. The former centers provide active aging programs and social support for community-dwelling older adults. The latter centers run programs to promote mental wellness in the community and provide psychosocial support for community-dwelling residents of all ages with mental health challenges. The research team developed a short survey instrument and trained center-based social workers and volunteers to conduct the survey. We called a total of 3550 members and completed 3421 interviews, giving a success rate of 96.4%. All respondents had no prior history of COVID-19.

### Data Collection

Data were collected through telephone interviews. Each interview lasted for about 10 to 15 minutes. The questionnaire was tested and revised before full-scale implementation. Qualified clinical psychologists and the researchers of this study designed the drafted questionnaire, which was reviewed and pretested by frontline social workers. Questions that were identified as challenging for older people with limited education to understand were revised and simplified. The process was repeated for two rounds before large-scale implementation. Interviewers were trained to make phone calls to respondents using a standardized, rapid, warm-call protocol. The protocol started with a warm call; that is, getting in touch with service recipients with known personal information. After greeting them and asking for their needs amid the pandemic, the questionnaire was fielded. Frontline social workers followed up with participants who demonstrated mental health challenges. This study was approved by the Human Research Ethics Committee of the University of Hong Kong (reference No. EA2003001[A]).

### Measurements

The major dependent variables of the study were anxiety, social trust in information, and COVID-safe behaviors. Anxiety was measured using the validated Chinese version of the 2-item Generalized Anxiety Disorder (GAD-2) scale, which ranged from 0 to 6, where a GAD-2 score of 3 or more indicated a high possibility of anxiety [35,36]. For social trust in information, respondents were asked to rate the extent to which they believed the COVID-19-related information that was shared from people in their social circles, including family, friends, and professionals (eg, social workers and general practitioners), on a scale from 1 (total distrust) to 5 (complete trust). The scale included a *not applicable* option that resulted in only 1480 respondents completing all three questions. Social trust in information was computed as the average of the three items for ordinary least squares (OLS) regressions (Cronbach α=.69) and as a latent variable of the three items in structural equation modeling (SEM). Only responses with complete answers in all three items were compiled. By treating social trust in information as missing when the respondents selected *not applicable* in any of the three components, the valid number of responses of social trust in information in OLS regressions was reduced to 1480. In the SEM, missing data were treated with full information maximum likelihood (FIML) estimation. The sample size for the SEM was 3415. COVID-safe behaviors were proxied by social distancing, measured by the older adults’ responses to how many days each week they ventured into the community.

The major independent variable was social media usage for COVID-19-related information, which was measured with three categories: (1) no usage, (2) social media used for COVID-19-related information, and (3) social media used as the main source of COVID-19-related information. Usage of social media for COVID-19-related information was recoded from a survey item on the trust level of media sources. The trust levels of social media and traditional media were captured in a 5-point scale, ranging from 1 (total distrust) to 5 (complete trust). The scale included a *not applicable* option. Only respondents using social media for COVID-19-related information were prompted to answer the question about trust level of social media, while nonusers picked the *not applicable* option. To measure any use of social media for COVID-19-related information, valid responses on social media trust were, therefore, recoded as *used social media for COVID-19-related information*. The nonusers who picked *not applicable* were considered to have no usage. The main information source was captured by a dichotomous variable of *traditional media* or *social media*, where the *social media* responses were recoded into *social media used as the main source of COVID-19-related information*.

The control variables include the risk of contracting COVID-19, depressive symptoms, and demographics. The risk of contracting COVID-19 was measured by the number of all confirmed cases in the week of data collection in the district in which the respondent lived. This information was obtained from the government daily report of newly confirmed cases. This measurement is geographically sensitive and reflected the risk of anyone contracting COVID-19, older adults included, who ventured into their neighborhood. Depression was measured using the validated Chinese version of the 2-item Patient Health Questionnaire (PHQ-2), with scores ranging from 0 and 6, where a PHQ-2 score of 3 or more indicates a high possibility of depression [37,38]. Demographics collected included respondents’ age in years, gender (ie, male or female), district of residency, and membership of service unit (ie, elderly center or mental wellness center). Respondents from mental wellness centers, who had a history of mental challenges, were included in the sample. A dummy variable was added to statistical models to control for its effects on mental health status and to investigate any difference in using social media for COVID-19 information.

### Statistical Analysis

Descriptive statistics of all variables were computed and reported as appropriate. Social media usage categories were entered into the statistical models as dummy variables of *used social media for COVID-19 information* and *used social media as the main COVID-19 information source*, with *no usage* as the reference group. Multivariate OLS regressions were applied to investigate the effect of using social media for COVID-19 information on anxiety symptoms as well as the effects of using social media for COVID-19 information and anxiety symptoms on time spent in the community and social trust in information. The theoretical model was then examined by SEM to test the mediation effect of anxiety symptoms. Social trust in information was treated as a latent construct with the three trust items. The first level of the SEM predicted anxiety symptoms with depressive symptoms, using social media for COVID-19 information, and demographics. The second level predicted time in the community and social trust in information with using social media for COVID-19 information, anxiety symptoms, and demographics. The FIML approach, which estimates parameters and standard errors directly with all data but does not impute missing data, was employed to account for the missing data in SEM. FIML generates relatively unbiased estimates and the least convergence failures compared to other methods, such as listwise or pairwise deletion and multiple imputations in SEM [39]. It is also less affected by nonnormal missing data and data distribution shape [40].

All data were consolidated and analyzed with SPSS software, version 26 (IBM Corp), after the removal of personal identifying information. SEM was conducted using the R package *lavaan* (The R Foundation) [41].

## Results

Table 1 shows the respondents’ characteristics. Their average age was 76 years (SD 8.9), 25.4% (869/3418) were male, and 77.9% (2666/3421) were recruited from elderly centers. The average number of COVID-19 cases in a community was 25.7 (SD 27.5), ranging from 0 to 135. Depression and anxiety were not prevalent: the average PHQ-2 score was 0.83 (SD 1.3) and the average GAD-2 score was 0.74 (SD 1.2). While 8.7% (298/3421) of the respondents exhibited a risk of having depression (ie, PHQ-2 score ≥3), 7.0% (241/3421) of the respondents exhibited a risk of having anxiety disorder (ie, GAD-2 score ≥3). Out of 3421 respondents, 1399 (40.9%) used social media to obtain COVID-19-related information and 203 (5.9%) used social media as their main source of COVID-19 information. The respondents had spent some time outside the home in the community on an average of 4 days (SD 2.4) in a week. In terms of their attitudes, the respondents had moderate to high levels of social trust in information (family: mean 4.4, SD 0.83; friends: mean 3.6, SD 0.96; professionals: mean 4.5, SD 0.74).

Compared to respondents recruited from mental wellness centers, respondents from elderly centers showed a different profile in their demographics and mental health status, but not in their usage of social media. Respondents from elderly centers were, on average, 4.4 years older (t_3419_=12.0, *P*<.001), and more of them were male (elderly centers: 703/2663, 26.4%; mental wellness centers: 166/755, 22.0%; χ^2^_1_=6.0, *P*=.01). They also showed fewer depressive symptoms as measured by the PHQ-2 (elderly centers: mean 0.69, SD 1.18; mental wellness centers: mean 1.33, SD 1.53; t_3416_=12.3, *P*<.001) and fewer anxiety symptoms as measured by the GAD-2 (elderly centers: mean 0.61, SD 1.13; mental wellness centers: mean 1.22, SD 1.43; t_3386_=12.1, *P*<.001). Nevertheless, there was no significant difference in their usage of social media (t_3419_=1.35, *P*=.18), which was the main independent variable. To account for the potential bias from a different mental health profile, the nature of the service was treated as a control variable in the subsequent analysis.

**Table 1:** Respondent characteristics.

Variable		Value (N=3421)
**Demographics**		
	Age (years), mean (SD)	76.0 (8.9)
	Gender (male) (n=3418), n (%)	869 (25.4)
	Service nature (aged care), n (%)	2666 (77.9)
**Community COVID-19 risk, mean (SD)**		
	Weekly number of COVID-19 cases in district	25.7 (27.5)
**Psychological well-being (range 0-6), mean (SD)**		
	2-item Patient Health Questionnaire (n=3418)	0.83 (1.3)
	2-item Generalized Anxiety Disorder scale (n=3388)	0.74 (1.2)
**Trust in media (range 1-5), mean (SD)**		
	Traditional media (n=3335)	4.27 (0.88)
	Social media (n=1399)	3.18 (1.1)
**Using social media for COVID-19 information**		
	Used social media for COVID-19 information, n (%)	1399 (40.9)
	Social media as the main source of COVID-19 information, n (%)	203 (5.9)
Days out in the community per week (n=3161), mean (SD)		4.1 (2.4)
**Social trust in COVID-19 information (range 1-5), mean (SD)**		
	Family (n=2620)	4.4 (0.83)
	Friends (n=2023)	3.6 (0.96)
	Professionals (n=2150)	4.5 (0.74)

Table 2 shows the results of the OLS regressions. After controlling for demographics and depressive symptoms, the category *used social media for COVID-19-related information* was not associated with anxiety symptoms but *used social media as the main source of COVID-19 information* did (B=0.18, *P*=.003). Using social media as the main source for COVID-19-related information was associated with more anxiety symptoms. Although social media use did not predict time spent in the community, it was associated with lower social trust in information. Both levels of usage—*used social media for COVID-19-related information* (B=–0.11, *P*=.005) and *used social media as the main source of COVID-19 information* (B=–0.30, *P*<.001)—showed a negative association. Anxiety symptoms were also negatively associated with time spent in the community and social trust in information (days out: B=–0.08, *P*=.02; social trust: B=–0.04, *P*=.02). Community COVID-19 risk was not significantly associated with anxiety symptoms (B=0.001, *P*=.69) but predicted less time spent in the community (B=–0.01, *P*<.001) and higher social trust in information (B=0.003, *P*<.001). Aged care respondents showed fewer anxiety symptoms (B=–0.17, *P*<.001) and higher social trust in information (B=0.23, *P*<.001), while older respondents spent the least amount of time in the community (B=0.03, *P*<.001). Male respondents were less anxious (B=–0.09, *P*=.004), spent more days out in the community (B=0.24, *P*=.02), and had lower social trust in information (B=–0.10, *P*=.02).

**Table 2:** Results from ordinary least squares regressions predicting anxiety symptoms, days out in the community, and social trust in information.

Variable	GAD-2^a^ scale (n=3384)			Days out in the community (n=3126)			Social trust in information (n=1480)	
	B^b^ (95% CI)	*P* value	B (95% CI)		*P* value	B (95% CI)		*P* value
Gender (male)	–0.09 (–0.15 to –0.03)	.004	0.24 (0.04 to 0.43)		.02	–0.10 (–0.18 to –0.02)		.02
Age (years)	–0.002 (0)	.19	–0.03 (–0.04 to –0.02)		<.001	–0.001 (–0.01 to 0)		.80
Community COVID-19 risk	0.001 (0)	.07	–0.01 (–0.01 to 0)		<.001	0.003 (0)		<.001
Service nature (aged care)	–0.17 (–0.24 to –0.10)	<.001	0.04 (–0.17 to 0.26)		.68	0.23 (0.14 to 0.32)		<.001
Depressive symptoms	0.71 (0.69 to 0.73)	<.001	N/A^c^		N/A	N/A		N/A
Used social media for COVID-19 information	0.002 (0 to 0.10)	.95	0.02 (–0.17 to 0.22)		.81	–0.11 (–0.19 to –0.03)		.005
Used social media as the main COVID-19 information source	0.18 (0.06 to 0.30)	.003	0 (–0.36 to 0.36)		.99	–0.30 (–0.43 to –0.16)		<.001
Anxiety symptoms	N/A	N/A	–0.08 (–0.15 to –0.01)		.02	–0.04 (–0.07 to –0.01)		.02

^a^GAD-2: 2-item Generalized Anxiety Disorder.

^b^Unstandardized coefficient.

^c^N/A: not applicable. The 2-item Patient Health Questionnaire (PHQ-2) was used for controlling the comorbidity with anxiety in the equation regressing GAD-2; there are no PHQ-2 values because the PHQ-2 was not included in the equations.

**Figure 2:**
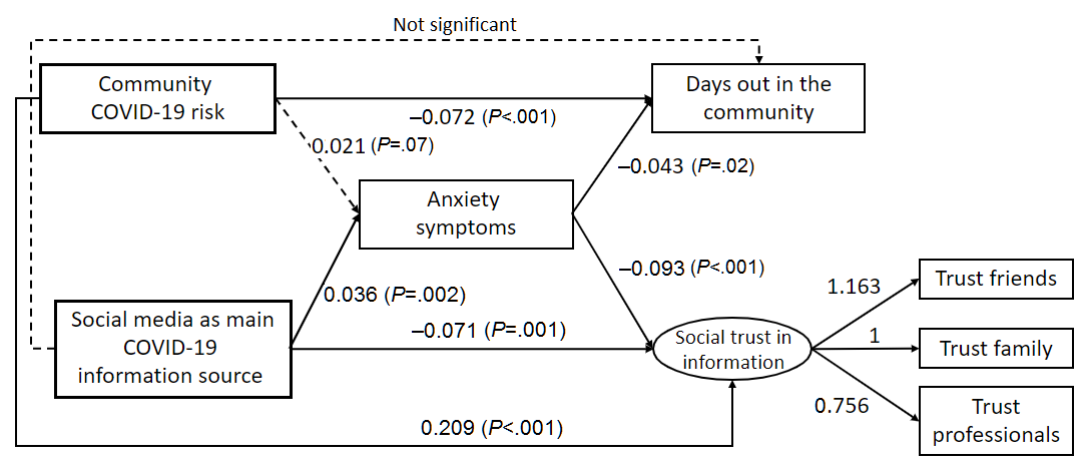
Structural equation modeling. Standardized parameter estimates are reported.

The results of SEM analysis (see Table 3 and Figure 2), modeling the mediation effect of anxiety symptoms, showed a good fit (χ^2^_19_=48.0, *P*<.001; Comparative Fit Index [CFI]=0.993; Tucker-Lewis Index [TLI]=0.985; root mean square error of approximation [RMSEA]=0.021; standardized root mean residual=0.014) and coincided with most findings in the OLS regressions. Since a lower level of social media use for COVID-19-related information was not associated with anxiety symptoms in the OLS regressions, the category was collapsed with *no social media usage* in the SEM. Using social media as the main source of COVID-19-related information predicted more anxiety symptoms (SEM coefficient=0.036, *P*=.002). Anxiety, meanwhile, was associated with lower social trust in information (SEM coefficient=–0.093, *P*<.001) and less time spent in the community (SEM coefficient=–0.043, *P*=.02). Using social media as the main source of COVID-19 information had a direct association with lower social trust in information (SEM coefficient=–0.071, *P*=.001), which means relying on social media for COVID-19 information was a significant predictor of social trust in information. Moreover, an indirect effect of using social media for COVID-19 information was mediated by anxiety (total effect=–0.074, *P*=.001). However, using social media as the main source of COVID-19 information was not associated with time spent in the community (SEM coefficient=–0.001, *P*=.97). No mediation effect was detected either (total effect=–0.002, *P*=.90). Sensitivity checking using social trust in information and time spent in the community to predict anxiety symptoms in SEM showed no significant associations (CFI=0.988; TLI=0.976; RMSEA=0.027), suggesting that anxiety was a valid independent factor in the second level of the original SEM model.

**Table 3:** Standardized parameter estimates and their standard errors of structural equation modeling (N=3415) treated with full information maximum likelihood estimation.

Equation model or regression			Standardized parameter estimate		SE		*P* value	
**Measurement model**								
	Social trust in information measured by trust in family			1		N/A^a^		N/A
	Social trust in information measured by trust in friends			1.163		0.064		<.001
	Social trust in information measured by trust in professionals			0.756		0.041		<.001
**Regression**								
	**Anxiety symptoms measured by the following:**							
		Age (years)		–0.017		0.002		.15
		Gender (male)		–0.032		0.032		.004
		Service nature (aged care)		–0.057		0.036		<.001
		Community COVID-19 risk		0.021		0.001		.07
		Depressive symptoms		0.740		0.011		<.001
		Social media as the main COVID-19 information source		0.036		0.060		.002
	**Social trust in information measured by the following:**							
		Age (years)		0.106		0.001		<.001
		Gender (male)		–0.062		0.030		.007
		Service nature (aged care)		0.138		0.032		<.001
		Community COVID-19 risk		0.209		0		<.001
		Social media as the main COVID-19 information source		–0.071		0.052		.001
		Anxiety symptoms		–0.093		0.011		<.001
	**Days out in the community measured by the following:**							
		Age (years)		–0.102		0.005		<.001
		Gender (male)		0.042		0.097		.02
		Service nature (aged care)		0.008		0.108		.69
		Community COVID-19 risk		–0.072		0.002		<.001
		Social media as the main COVID-19 information source		–0.001		0.176		.97
		Anxiety symptoms		–0.043		0.035		.02

^a^N/A: not applicable; this is the reference variable.

## Discussion

### Principal Findings

Our findings are not only consistent with previous studies on the association between social media use and anxiety [8,9], especially during the COVID-19 pandemic [11-13], but they also identified the major type of social media use among older adults (ie, consuming information) as predicting more anxiety symptoms. Both the OLS regression and SEM results demonstrated the association between using social media for COVID-19-related information and more anxiety symptoms among older adults. On the one hand, the ecology of social media, characterized by information overload and sensationalizing of information [42,43], could have amplified older users’ nervousness and perceptions of the risks of contracting COVID-19. The inconsistency in unverified information on social media exacerbates this anxiety. On the other hand, older people’s passive usage of social media suggests an intensified anxiety [9,10]. Since mental well-being decreases when social media users fail to acquire direct social interaction to satisfy their relatedness needs [44,45], passive usage may intensify unmet needs resulting from social isolation during the pandemic, thus prompting anxiety symptoms.

The SEM results suggested that anxiety symptoms mediated the effect of using social media for COVID-19 information on social trust in information. It demonstrated the two pathways of the associations between using social media and attitudes in trusting information. The proliferation of unverified and emotional information may drive anxiety and lower social trust in information among social media users. Algorithms in social media platforms prioritize sensational information [43]. User attention is drawn to the emotions induced while diminishing the ability to critically evaluate the accuracy of information, promoting the impulsive spread of unverified misinformation [46]. As a result, social media users may face contradictory information on social media, which may also be inconsistent with the information acquired from family, friends, or even health professionals. Not only would they become anxious about what to believe, but social trust in information was also undermined by reading conflicting claims. Another problem brought by social media use is the overwhelming amount of information overloading users as a result of uncontrolled news consumption [42]. On popular social media platforms, such as Facebook and Twitter, an infinite scroll feeds content to users without a “stopping cue” [47], while algorithms tailor content that suits user preference [43]. The environment in social media suggests that users are encouraged to stay on their sites as long as possible, consuming more information than intended. Overload is further magnified by the challenge to process the unverified, anonymous, and overwhelmingly subjective news on social media [48]. Consequently, it is difficult to digest all the information flooding into the social media feed because of both its enormous quantity and questionable quality.

Differences were observed from various demographic profiles as well. Older age was associated with spending fewer days venturing into the community, which was likely related to the lowered mobility in aged respondents. Male respondents were less anxious and more critical of COVID-19 information from people around them, and they went into the community more often. The results may have demonstrated a more authoritative gender role in older East Asian men. Respondents from mental wellness centers showed more anxiety symptoms and lower social trust in information. This suggested that a history of mental health challenges may exacerbate the effects of the infodemic.

Our results demonstrated that the infodemic may have caused more confusion than promoting COVID-safe behaviors. While older adults’ usage of social media for COVID-19 information showed no association, the number of COVID-19 cases in the community and anxiety symptoms predicted less time spent in the community, suggesting that older adults adopted COVID-safe behaviors when the risk was high and when they became anxious. COVID-safe behaviors may have been further encouraged by the surge of social distancing and stay-home advice when the number of confirmed cases grew. However, these messages might not be transmitted equally to all social media users. Health-related misinformation proliferated well before the pandemic, facilitated by “information silos” and “echo chambers” in social media [49]. The clustering of users in social media means that both useful information and harmful misinformation may not reach a mass audience but is amplified within social bubbles. Different stories could be proliferating among different user subgroups, resulting in the uneven effects of consuming information on social media regarding COVID-safe behaviors.

A greater understanding of how anxiety affects behaviors could inform ways to communicate COVID-19-related information to older adults to promote appropriate COVID-safe behaviors and social trust in information and contribute to the wider public health effort. On the one hand, our results suggested that anxiety encouraged older adults to avoid social contact. Nevertheless, more research is warranted to understand if this is rational risk aversion or panic-induced maladaptive behavior. On the other hand, while it is normal to be anxious in the face of a global pandemic, future research should also focus on the management and mitigation of anxiety during a prolonged pandemic so that older adults’ trust in the people around them, who may provide the most adequate and timely support, is less likely to be undermined.

Although this study suggests some adverse effects of using social media, it does not mean that older adults should avoid using it. On the contrary, it is necessary to address these problems and identify the potential benefit of using social media to promote adequate COVID-safe behavior and reduce loneliness amid social distancing. Digital skills alone may no longer be sufficient to enable older adults to navigate the complex contemporary media environment. Media literacy among older adults should be advocated to enable them to critically analyze and interpret information. Guidance should be provided to promote healthy coping behaviors, such as verifying suspicious information and searching for credible information sources in response to the infodemic in social media. Advice on responsible social media use is essential for building a constructive social media environment for older adults. Media literate older adults should understand the consequences of spreading misinformation and refrain from sharing unverified information. By empowering older adults to distinguish and reject misinformation, the infodemic may be alleviated if the transmission of misinformation is curbed in its early stages. Anxiety may be reduced when older adults acquire agency while mutually building a healthy social media environment.

### Limitations

Since the rapid warm-call protocol was designed to conduct interviews quickly with an extensive reach, our questions were designed to be simple and concise. While the data collection period spanned across 4 months, the study was cross-sectional in nature, which could not allow it to demonstrate causal relationships between use of social media for COVID-19, social trust, and anxiety. The self-reported survey design may also suffer from data inaccuracy as a result of issues including social desirability bias or loss of memory. Although a measurement for social media usage for COVID-19 information was constructed for this study, the sensitivity of the measurement may have been compromised for the conciseness of the survey. The range in measuring *used social media for COVID-19 information* may be too wide to fully capture its pro rata effects. On top of that, the frequency of media use is yet to be addressed. The frequency or time spent on social media may provide greater information about the influence of media usage patterns on anxiety, attitudes, and behaviors. Our scale did not capture the variance in the amount of time exposed to social media, only its ratio. Therefore, the study may underestimate the effect of using social media for COVID-19 information. Meanwhile, only one COVID-safe behavior—staying home to avoid social contact—was measured. Potential bias may occur when other factors contribute to older adults staying at home, such as the closure of businesses and social services, that were unrelated to using social media for COVID-19 information and anxiety.

Our sample, recruited from both elderly and mental wellness centers, was not a representative sample of the city. The findings may not be generalized to the whole population. Without support from social service units, the unsampled cluster of older adults could have experienced a greater challenge in navigating the pandemic. They might exhibit greater anxiety from not receiving adequate social support, and social media might be a more significant source of information about the COVID-19 pandemic.

### Conclusions

This study suggests that using social media for COVID-19 information by older adults in Hong Kong during the COVID-19 pandemic was associated with anxiety symptoms. Meanwhile, anxiety symptoms mediated the effect from using social media for COVID-19 information on social trust in information but not COVID-safe behaviors. This suggests that the infodemic may have caused confusion when older adults digested health information, prompting anxiety among them about what and who to believe and, thus, reducing social trust in information. The fragmentized social media landscape further promoted an uneven impact of the infodemic between different groups of users. It could explain why objective risk and anxiety predicted older adults’ avoidance of leaving home but using social media for COVID-19 information did not. To encourage the healthy use of social media, media literacy education should be promoted among older adults.

## Abbreviations

CFI: Comparative Fit Index
FIML: full information maximum likelihood
GAD-2: 2-item Generalized Anxiety Disorder
MERS: Middle East Respiratory Syndrome
OLS: ordinary least squares
PHQ-2: 2-item Patient Health Questionnaire
RMSEA: root mean square error of approximation
SEM: structural equation modeling
TLI: Tucker-Lewis Index
